# Room corners and how they influence the memory of visual information arranged on walls

**DOI:** 10.1038/s41598-024-62648-1

**Published:** 2024-05-26

**Authors:** Bärbel Garsoffky, Stephan Schwan

**Affiliations:** Leibniz-Institut für Wissensmedien, Tübingen, Germany

**Keywords:** Human behaviour, Psychology and behaviour

## Abstract

In formal and informal learning settings corresponding information units are often arranged in the space around the viewer. For example, teachers pin task relevant information on classroom walls, or museum curators arrange exhibits in museum halls. Often learners and visitors are expected to see meaningful relationships between these information units. Theoretically, Gestalt Psychology has been examining the effects of connecting and separating elements in visual information displays, leading to the question of whether these findings also hold in three-dimensional environments. Does the mostly rectangular form of our rooms also either highlight or downplay relations between information dispersed across a room? Three experiments using virtual rooms showed that the matching pairs of pictures were memorized better if both pictures were arranged on the same wall instead of across two adjacent walls: that is, the presence of a room corner between matching pairs decreased memory (Experiments 1–3). Additionally, the findings showed that the participants’ orientation of their central field of view during learning influenced the effect of corners on memory. When initially looking around in rooms, participants most often oriented the center of their field of view toward the middle of a wall (Experiments 1 and 2); however, if they were restricted to orienting their field of view toward corners, the corner effect on memory vanished (Experiment 3). These findings suggest that room characteristics influence the exploratory behavior of viewers, thereby also affecting their memories of the presented information.

## Introduction

In the movie “The Name of the Rose”^1^, the labyrinthine architecture of a medieval library is used to hide dangerous information from users. However, architecture may also support people in processing information units and their interrelations. However, how can this be done precisely? In everyday life, we often spend much time in buildings. The rooms become the background to the places where we live and work. We are used to being in rooms, and therefore we may think of them as neutral and merely a physical necessity. However, theories and findings from very different areas of psychological research suggest that the characteristics of rooms influence not only emotion and mood but also basic human cognition. One basic feature of nearly every room in a built environment is corners, which in turn define walls. Furthermore, there are many rooms in which information is arranged across several walls, for example in formal and informal learning settings such as schools and museums but also in commercial environments such as retail stores.

The question arises, do corners influence the processing of information arranged on walls? In the following, three experiments are presented that examine the influence of room corners on memory for visual picture pairs distributed on walls. A matching pair was formed by two identical pictures presenting an everyday object. Do people remember related information better when parts of the information, in this case the two pictures constituting a matching pair, are arranged on the same wall instead of across two adjacent walls separated by a corner?

In particular, Gestalt Psychology addresses the connection between how visual information is spatially arranged and how it is perceived and processed. Palmer^2^ stated that stimuli are grouped during perception and that simple line contours around stimuli can induce the perception of common regions and therefore grouping, as well as the same brightness, proximity, or connecting lines between stimuli. Another study examining grouping principles revealed that pictures of a pair were detected faster when they belonged to the same group or common region^3^. The present experiments extend the findings of common regions and examine whether common regions can also be found in virtual three-dimensional room environments.

McNamara^4^ discussed basic concepts of spatial mental models and distinguished nonhierarchical and hierarchical models by investigating whether spatial relations between information units are only cognitively represented within the subregion they are displayed (strongly hierarchical models) or also across the borders of subregions (nonhierarchical models). He found that an object acted as a stronger prime if it was in the same region as the target object and if it was nearer to the target object, thereby supporting the idea of a partially hierarchical model. The purpose of the following experiments is to further refine this approach by examining whether spatial visual arrangements are still processed in a partially hierarchical way if the experimental task explicitly demands that participants ignore boundaries of subregions and to mentally relate information units across these borders.

In addition to extending and refining basic theoretical concepts such as common region^2^ and spatial mental models^4^, the question of whether the characteristics of our everyday environment influence cognition has become increasingly important for research on learning and working environments. Choi, van Merrienboer and Paas^5^ extended their model of cognitive load by systematically discussing empirical findings concerning learning in formal and informal learning settings. The extended model postulates that not only “task” and “learner” fundamentally determine the cognitive load that emerges in a learning situation but also “environment”. In line with this argument, several studies have shown that basic architectural characteristics of working spaces such as ceiling height^6^, round versus angular form^7,8^ or room doors^9^ and size^10^ influence specific kinds of cognitive processes such as relational vs. item-specific processing, convergent vs. divergent thinking, accessibility of information in situation models, or effort allocation on the task at hand.

In addition, when spatially arranging information, the distance between stimuli must be considered. The findings suggest that the semantic relation and the spatial distance between information units mutually depend on each other: Semantically related objects are remembered with smaller spatial distances between them than objects that are not semantically near^11,12^, and two objects that are spatially nearer together more often are named with a common generic categorical term than two objects spatially farther away from each other^13^.

## Experiments and results

Three experiments examined the influence of room corners on memory for pairs arranged on the walls of a virtual room. It was expected that corners would lead to the perception of common regions, thereby dividing the room into subregions, which in turn would lead to better memory for matching pairs if the two pictures were presented on the same wall instead of being arranged across two adjacent walls. Furthermore, it was examined whether the distance between two pictures influenced the memory of the pairs.

All three experiments were conducted online (https://www.prolific.co) and participants took part in each of the experiments using their own hardware, either laptops or computers. In all three experiments, the participants saw a virtual room (programmed with Unity) and looked and turned around in the room presented on their monitor by pressing the right and left arrow keys. They played a variant of the matching pairs memory game: On the walls of the virtual room, picture pairs of everyday objects (for example, two times exactly the same picture of an apple) were arranged. Each participant worked through eight rooms containing 36 picture cards (i.e. 18 pairs), arranged across three walls, with two rows of 6 cards each on every wall, and each room related to one semantic category (e.g. fruits, sweets, clothing, vehicles), each followed by twelve trials in the test phase.

The rooms had a four sided floor, and the walls were rectangular (width: height = 2.25: 1). Because the viewers were fixed to the center of the room and could only turn around, but not walk, it could be controlled, that at each point of time a participant had 6 pictures next to each other in his or her field of view (in two rows with 6 pictures each). This was the case if a participant looked in the middle of a wall, but also if he or she looked, for example, straight towards a corner. So, when looking towards the middle of a wall, all pictures on that wall were within the field of view, and the same number of pictures was visible when looking towards a corner. The metric distance between two pictures was the same, independent if there was a corner between the two pictures or not: In the case of a corner between the two pictures, the distance was measured by a thought line “through the air”, not along the walls. So, the metric distance was not greater if the two pictures were arranged on two adjacent walls than if they were arranged beneath each other within one wall.

**Figure 1:**
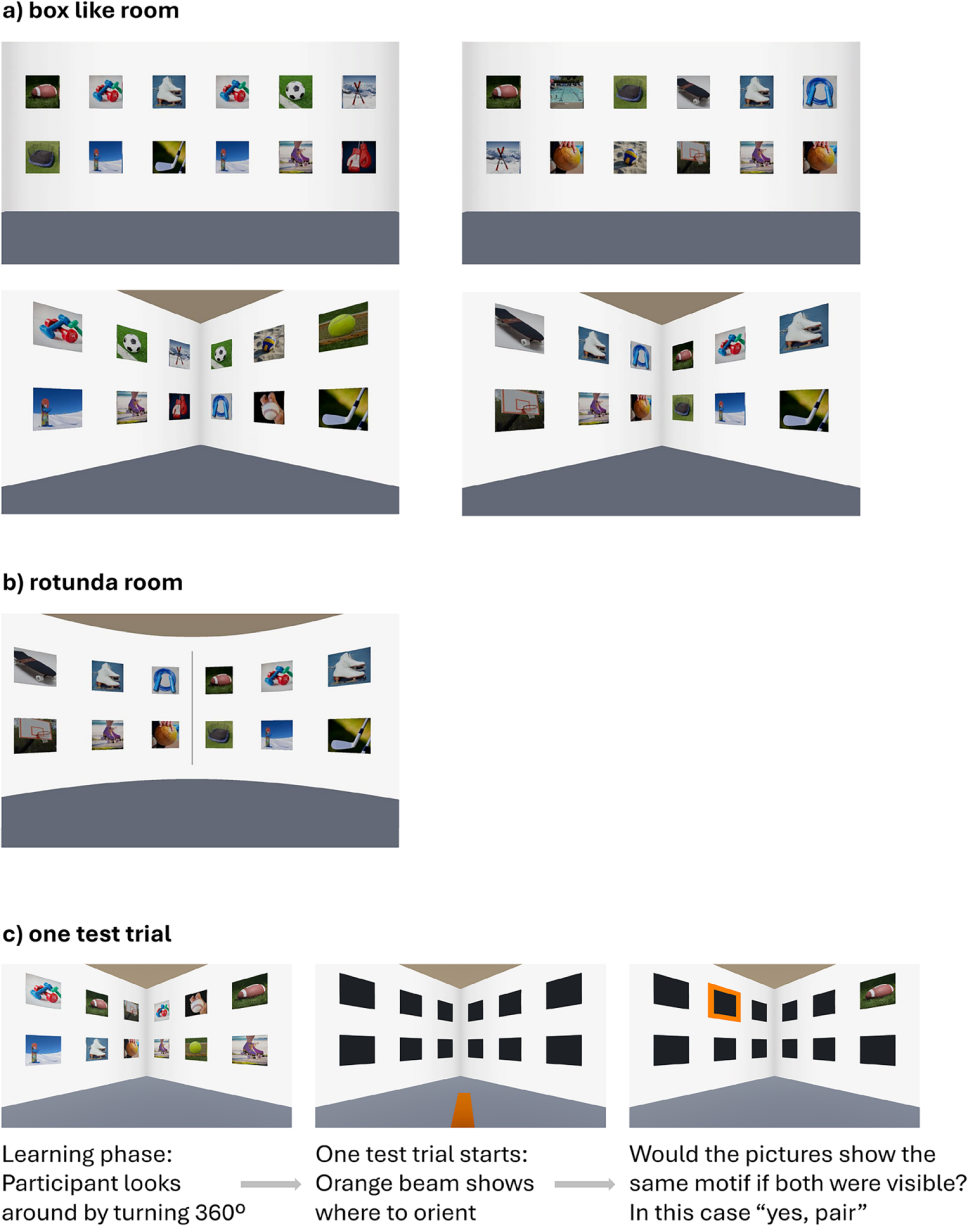
Experimental material in Experiment 1–3: (**a**) Examples of the box room in the learning phasse in the upper row from left to right: two pictures forming a pair within one wall and one other picture between (snowboard), one wall and three between (bowling), and in the lower row across two walls and one between (soccer ball), across two walls and three between (ice skates). (**b**) One example of the rotunda room, in this case a pair arranged across two sides of the line (ice skates). (**c**) Shows one test trial in the test phase: In this example the trial tested two pictures on different walls with 3 pictures between them (football). The correct answer was “A” for “pair” because the two pictures showed the same subject (in this example, football). During the learning phase, participants had the impression of standing in the middle of the virtual room and could turn around by using the left and right arrows on their keyboard (rotation) either freely (Experiment 1 and 2) or in steps of 90 degrees (Experiment 3), but they could not move through the room (translation). During the test phase they could turn around freely in all three Experiments.

First, in the learning phase, all picture pairs in the room were simultaneously visible. The participants were asked to remember the position of all the pictures by looking around in the room for two minutes and were instructed to memorize the pairs. In the training block (as well as later in the experimental blocks) the pairs were arranged equally often within the same and across two adjacent walls. Therefore, it was explicitly trained to memorize pairs independent from their distribution across walls, but this was not formulated in the instructional text, to avoid making the experimental variation too obvious and thereby to avoid that participants guess the hypothesis. A good performance simply required to remember pairs arranged within one as well as across two walls. Then, during the test phase, the participants saw the same virtual room again, but now all the picture cards were turned so that only their blank backs were visible. Next, in each test trial one card was uncovered so that the picture became visible and a second card with only its blank back visible was marked by an orange frame. The participants had to decide if these two cards would show the same picture if the second card would be uncovered. There was no temporal restriction during the test phase. For an example of the virtual room and the procedure used in the test trials, see Fig. 1.

The arrangement of the picture cards realized the factors wall (if the matching pairs were arranged within one vs. across two adjacent walls) and distance (there was one vs. three other picture cards between the two matching pictures forming a pair). Importantly, wall and distance were varied independently in a within-subjects design, i.e. half of the pairs that were later used for the test trials were arranged within one wall and the other half of them across two walls. Half of these pairs later used for test trials and that were arranged within one wall had one other picture card between them and the other half of them had three picture cards between, and the same was true for pairs arranged across two walls. Additionally, in each room four pairs were presented in different rows to avoid participants to memorize the arrangements in a row-wise manner. Otherwise, all other picture cards were placed randomly.

It was measured, how good participants were in memorizing pairs by using the sensitivity A’ measure (according to Macmillan & Creelman^14^ and Stanislaw & Todorov^15^. Based on considerations of signal detection theory^14^, when measuring memory of formerly learned material, it is not sufficient, to only count how often an old (learned) stimuli is truly recognized as “old” (a hit), but also to look how often a new stimuli (i.e. one that has not been learned before) is wrongly answered as being “old” (a false alarm). Otherwise, participants could show good performance in recognizing “old” stimuli simply by always hitting the key for “old” and not differentiating between “new” and “old” items at all. To consider that, sensitivity A’ is calculated by a formula (see Stanislaw & Todorov^15^ using not only the hit rate but also the false alarm rate, thereby measuring the ability of the participants to differentiate between “new” and “old” items. For exploratory reasons, during the learning phases in Experiments 1 and 2, every half second, it was recorded where they oriented their central field of view in steps of 360 degrees. For an overview of the results on memory (sensitivity) found in Experiments 1–3 see Table 1.

**Table 1:** Overview of significant effects.

	*Experiment 1* Architecture: box Viewing direction in learning phase: free	*Experiment 2* Architecture: box vs. rotunda Viewing direction in learning phase: free	*Experiment 3* Architecture: box Fixed viewing direction in learning phase: wall vs. corner
One wall vs. two (W)	One > two	One > two	One > two
Distance 1 vs 3 between (D)	1 > 3 between	1 > 3 between	1 > 3 between
W x D	n.s	n.s	1 between: On one = across two 3 between: On one > across two
Architecture box vs. rotunda (A)		n.s	
W x A		n.s	
D x A		n.s	
W x D x A		n.s	
Fixed viewing direction corner vs. wall (V)			n.s
W x V			Fixed view wall: One > two Fixed view corner: One = two
D x V			n.s
W x D x V			n.s

Overview of all significant effects found in the ANOVAs calculated in the three experiments showing results for memory (sensitivity A’). In the first column are the factors of the ANOVAs. For details of the effects, please see the text.

### Experiment 1: the corner effect on memory

**Figure 2:**
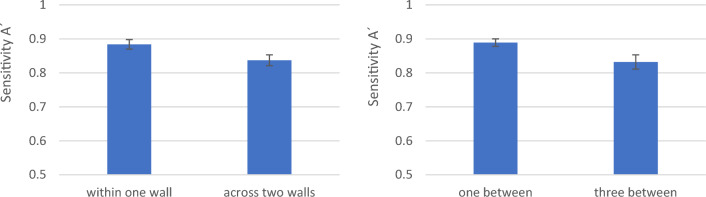
Main effects of wall (left) and distance (right) on sensitivity A’ in Experiment 1; error bars indicate standard errors.

The nonparametric sensitivity A’^13,14^ shows the ability to differentiate between pairs and no-pairs in the memory task. A two-factorial repeated measures analysis of variance (ANOVA) of A’ sensitivity with the factors “wall” (within one vs. across two) and “distance” (1 vs. 3 pictures) was conducted and revealed a significant effect for wall (F(1, 26) = 11.145, MSE = 0.005, p = 0.003, η_p_^2^ = 0.3) (see Fig. 2), showing that sensitivity was higher when the two cards of a test trial were on the same wall (M = 0.884, SE = 0.014) than when they were on different walls (M = 0.837, SE = 0.016). Additionally, distance had a significant effect (F(1, 26) = 8.032, MSE = 0.011, p = 0.009, η_p_^2^ = 0.236) (see Fig. 2). Sensitivity was higher if there was one card between the pictures of a pair (M = 0.889, SE = 0.011) than if there were three cards between them (M = 0.832, SE = 0.021). The interaction effect of both factors was not significant (F(1, 26) = 0.819, MSE = 0.008, p = 0.374, η_p_^2^ = 0.031).

**Figure 3:**
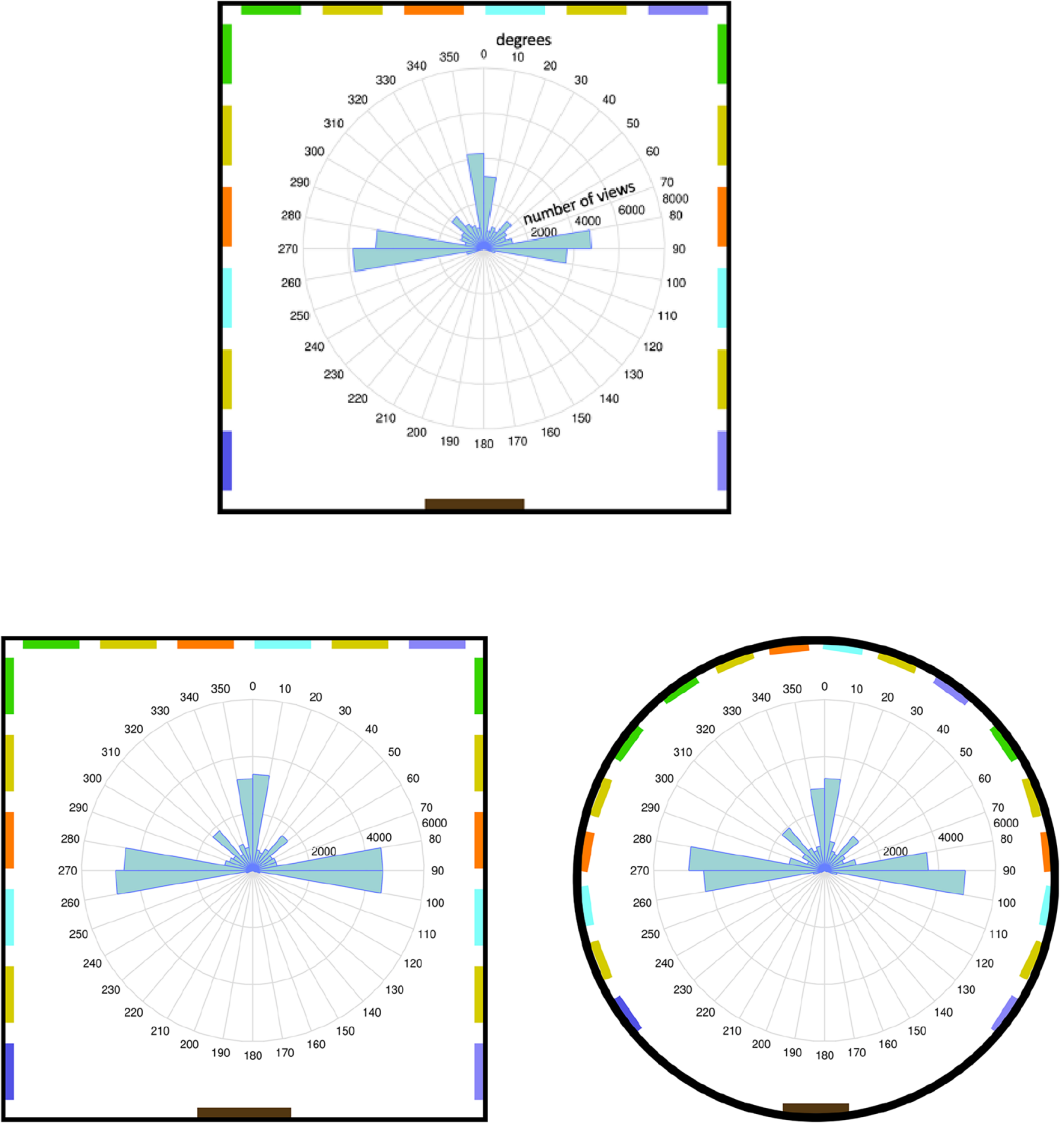
Number of participants’ viewing directions in 10-degree bins, measured every half second during the learning phase in Experiment 1 (boxroom, on the top of the figure) and Experiment 2 (box and rotunda room, bottom of the figure). The data display is surrounded by a rectangle/circle to visualize how the 360 degrees are mapped into the virtual room. The colored bars on the three walls symbolize the pictures, and the black symbolizes the door on the fourth wall of the virtual room. As shown, the central viewing directions were not distributed evenly throughout the room but were concentrated on the middle of the three walls with pictures.

Exploratorily, participants preferred to orient themselves facing the middle of walls instead of facing directly toward corners (descriptive presentation of the data in Fig. 3).

Taken together these findings suggest that participants memorized the visual information arranged in the room wall by wall, although they knew from the training block that matching pairs were arranged equally often within one wall and across two walls. Thus, there was a strong effect of a basic architectural feature, namely, a corner, on cognitive processing.

### Experiment 2: replicating the corner effect and examining lines

In addition to the four sided virtual room (i.e. a box room) used in Experiment 1, Experiment 2 also presented each participant with four learning rooms with a round floor (i.e. a rotunda room; see Fig. 1). Round rooms, of course, do not have any corners, and in Experiment 2 the round walls of these rooms were divided into four separate regions of the same size by perpendicular lines. It was examined whether simple lines have the same effect on memory as corners. Findings from Beck and Palmer^16^ and McNamara^4^ already indicated, that simple oval lines^16^ or ropes on the floor^4^ could also act as separators between information units.

A three-factorial repeated measures analysis of variance (ANOVA) of sensitivity A’ with the factors “architecture of room” (box vs. rotunda), “wall” (within one vs. across two) and “distance” (1 vs. 3 pictures) revealed a significant effect for wall (F(1, 50) = 15.356, MSE = 0.025, p < 0.001, η_p_^2^ = 0.235) showing that sensitivity was higher when the two cards of a test trial were on the same wall (M = 0.818, SE = 0.016) than when they were on different walls (M = 0.756, SE = 0.019). There was also a significant effect for “distance” (F(1, 50) = 17.486, MSE = 0.019, p < 0.001, η_p_^2^ = 0.259), with higher sensitivity if there was only one card between the two cards (M = 0.816, SE = 0.015) than if there were three cards in between (M = 0.759, SE = 0.018). There were no other significant effects or interactions in this analysis: “Architecture” (F(1, 50) = 2.958, MSE = 0.017, p = 0.092, η_p_^2^ = 0.056); “architecture” by “wall” (F(1, 50) = 0.122, MSE = 0.017, p = 0.728, η_p_^2^ = 0.002); “architecture” by “distance” (F(1, 50) = 1.075, MSE = 0.021, p = 0.305, η_p_^2^ = 0.021); “wall” by “distance” (F(1, 50) = 1.447, MSE = 0.016, p = 0.235, η_p_^2^ = 0.028); “architecture” by “wall” by “distance” (F(1, 50) = 0.151, MSE = 0.015, p = 0.699, η_p_^2^ = 0.003).

Exploratorily, participants preferred to orient themselves facing the middle of walls (for walls separated by corners as well as by lines) instead of facing directly toward corners or lines (descriptive presentation of the data in Fig. 3).

Taken together the findings replicated the results of Experiment 1 and showed that not only corners, but also lines between two pictures forming a pair had a detrimental effect on memory.

### Experiment 3: controlling the orientation of the central field of view during learning

Experiment 3 was conducted to investigate whether the corner effect of memory is influenced by the looking behavior of the participants. Exploratory findings in Experiments 1 and 2 showed that during learning participants preferred to orient their central field of view toward the middle of walls instead of toward corners. They did so, although the task required to memorize matching pairs arranged within one wall equally often as dispersed across two walls, and they did so although viewing directions toward walls and toward corners delivered the same amount of visible information, i.e., the same number of visible picture cards. Therefore, in Experiment 3, the orientation of the central viewing direction during learning was controlled: Participants could no longer freely turn around during the learning phase, as in Experiments 1 and 2, but they could only shift their field of view in steps of 90 degrees. In half of the rooms, each participant started facing the middle of a wall (thereby a shift of 90 degrees always centered the field of view toward the middle of another wall); in the other half of the rooms, each participant started facing toward a corner (thereby, a shift of 90 degrees centered the field of view always straight toward a corner). During the test phase, participants could freely turn around degree wise.

**Figure 4:**
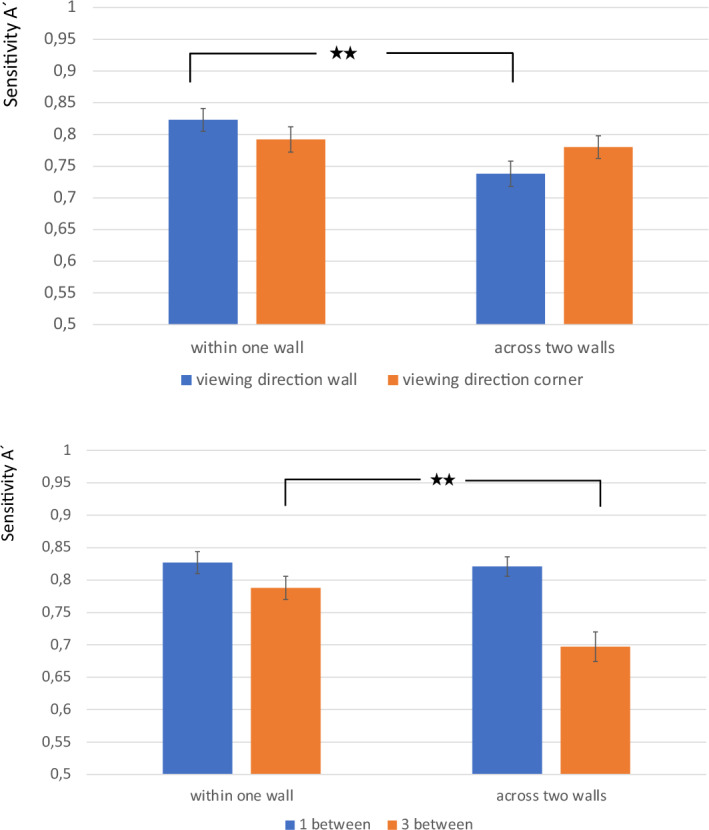
Interaction of wall and viewing direction on sensitivity A’ (top) and interaction of wall and distance on sensitivity A’ (bottom) in Experiment 3; error bars indicate standard errors.

A repeated measures analysis of variance with the factors “wall” (within one vs. across two), “distance” (1 vs. 3 pictures), and “fixed viewing direction” (centered toward wall vs. corner) again showed main effects of wall and distance on memory comparable to those of Experiments 1 and 2 but, more interestingly, also two interactions. The main effect of wall (F(1, 45) = 17.176, MSE = 0.012, p < 0.001, η_p_^2^ = 0.276) showed higher sensitivity if the two cards of a test trial were located within one wall (M = 0.807, SE = 0.015) instead of across two walls (M = 0.759, SE = 0.016). For the interaction between wall and viewing direction (F(1, 45) = 6.464, MSE = 0.019, p = 0.015, η_p_^2^ = 0.126) (Fig. 4), Bonferroni-adjusted post hoc analysis revealed that the difference between two cards arranged across two versus within one wall became significant only for the viewing direction towards the middle of the walls (MDiff = 0.085, SE = 0.019, p < 0.001, 95%-CI(0.046, 0.124)). If the participants’ viewing direction was centered toward the middle of the walls, sensitivity A’ was significantly higher for two cards arranged within one wall (M = 0.823, SE = 0.018) than across two walls (M = 0.738, SE = 0.020). However, if the participants’central viewing direction during learning was centered towards the corners, there was no significant difference in sensitivity between two cards arranged within one wall (M = 0.792, SE = 0.020) or across two walls (M = 0.780, SE = 0.018).

Furthermore, there was a significant main effect of distance (F(1,45) = 34.027, MSE = 0.018, p < 0.001, η_p_^2^ = 0.431), with sensitivity being higher if there was only one card between the two cards (M = 0.824, SE = 0.014) compared to three cards in between (M = 0.743, SE = 0.018).

Additionally, the interaction effect between distance and wall became significant (F(1,45) = 13.243, MSE = 0.012, p = 0.001, η_p_^2^ = 0.227) (Fig. 4). Bonferroni-adjusted post hoc analysis revealed significantly higher sensitivity A’ for two cards arranged on the same wall (M = 0.788, SE = 0.018) than for two cards arranged across two walls (M = 0.697, SE = 0.023) only if they were separated by three other picture cards in between (MDiff = 0.09, SE = 0.019, p < 0.001, 95%CI (0.052, 0.129). However, if there was one card between the two cards, sensitivity did not differ between arrangements within one (M = 0.827, SE = 0.017) or across two walls (M = 0.821, SE = 0.015). The effect of distance was significant for two cards arranged within one wall (MDiff = 0.39, SE = 0.016, p = 0.022, 95%-CI (0.006, 0.072)) as well as across two walls (MDiff = 0.124, SE = 0.02, p < 0.001, CI (0.084, 0.163)). There were no other significant effects or interactions in these analyses on sensitivity: “fixed viewing direction” (F(1, 45) = 0.132, MSE = 0.023, p = 0.718, η_p_^2^ = 0.003); “fixed viewing direction” by “distance” (F(1, 45) = 0.079, MSE = 0.017, p = 0.781, η_p_^2^ = 0.002); “fixed viewing direction” by “distance” by “wall” (F(1, 45) = 0.64, MSE = 0.025, p = 0.428, η_p_^2^ = 0.014).

Taken together, only if participants explored the picture cards in the learning phase viewing around wall by wall, did they memorize matching pairs arranged across two walls worse than matching pairs arranged within one wall. If participants learned the arrangement with their central field of view directed toward corners, it made no difference if matching pairs were arranged within one instead of across two walls. Thus, the effect of corner on memory found in Experiments 1 and 2 seems to depend on the looking behavior during the learning phase. Furthermore, the interaction between arrangement and distance showed that the effect of corner on memory was significant only if the matching pairs were separated by three cards instead of one card. This runs counter to one of Palmer’s conclusions^2^, namely, that common region should be more powerful for grouping than proximity is. In contrast, in Experiment 3, it seemed that if proximity was high (only one picture between), the common region (namely, if stimuli were arranged on either one or two walls) played no role. Only if the proximity was low (3 pictures between), did the common region become important, and memory was better if the stimuli were arranged within one wall instead of being distributed across two walls.

## Conclusion

The findings suggest that memory for visual information is influenced by the arrangement of related information either on the same or across two adjacent walls of a room (for an overview of the results on sensitivity, see Table 1), and that this effect is connected at least partially to the orientation of the visual field of view during learning: Experiment 1 showed that matching pairs were remembered better if they were arranged on the same wall of a virtual room than if they were arranged on two adjacent walls, thus revealing the effect of corners on memory. Experiment 2 replicated this corner effect on memory and, by introducing a rotunda room, additionally found that also lines on the wall of a round room decreased memory for matching pairs arranged on different sides of a line.

Concerning the viewing behavior of the participants during the learning phase, exploratory findings also suggested that if participants could freely choose where to orient the center of their field of view (Experiments 1 and 2), they preferred to orient it toward the middle of a wall instead of toward a corner. To examine the role of view orientation during the learning phase on the corner effect, Experiment 3 restricted participants' central orientation of their field of view during the learning phase to either the corners or the middle of walls. Again, there was a corner effect on memory, but only if participants were forced to orient their central viewing axis toward the middle of the walls while learning. The corner effect in Experiment 3 vanished if participants were forced to look directly toward corners while they were exploring the rooms.

In addition to this corner effect on memory, all three experiments showed the significant influence of distance on sensitivity; that is, matching pairs were memorized better if the two pictures were separated by one card instead of three cards.

In summary, the findings suggest that room corners have a detrimental effect on memory when related information is dispersed across different walls and that this may be caused by the participants’ preferred orientation of their central field of view toward the middle of walls: If participants can freely look around like in everyday situations, they prefer to orient their field of view more often towards the middle of walls than towards corners (see exploratory data on viewing behavior in Experiment 1 and 2). In this case, information dispersed within one wall more often is in the same visual field than information dispersed across two walls. And this in turn may explain why in Experiment 1 and 2 pairs were memorized better, if the two pictures were arranged within one wall instead of being arranged across two walls. Experiment 3 supported this consideration with the significant interaction between wall and viewing direction: If participants during learning were forced to orient their field of view towards the walls, there still was the corner effect of Experiment 1 and 2, namely, pairs were remembered better if they were arranged within one wall instead of on two walls. But if participants during learning were forced to orient their field of view towards the corners this advantage of being arranged within one wall was no longer significant; there was no significant difference between memory for pairs within one wall compared to pairs arranged across two walls. Interestingly, this non-significance between pairs within one and across two walls in the case when participants were forced to look towards the corners during learning gives a first hint that there may be more underlying the corner effect than only the field of view: If only the field of view would determine, which pairs are remembered better, than in the corner viewing condition there should have been an advantage for pairs across two walls over pairs within one wall, because they more often were seen together in the same visual field. But, of course, the fact that there is no significance can only be a first hint and should be replicated before being interpreted reliable. Further, future studies should take into account that the pictures were only arranged on three walls of the rooms. In Experiment 3 this led to the fact that, to see all pictures, participants in the wall-centered viewing condition during learning had to use three possible viewpoints (the three walls with pictures). In contrast, participants in the corner-centered condition had to use all four possible viewpoints during learning to see all pictures. But two of these possible corner viewpoints only displayed pictures on one side of the corner, i.e. two corner viewpoints displayed only half as much pictures as the other two corner viewpoints. Of course, participants knew that they had to look on all pictures to perform the memory task afterwards, but nevertheless it is possible that they looked more often or for longer duration towards the corners that displayed pictures on both side. And because the experimental stimuli pairs that were arranged across two walls were all presented in these two corners, this could also add to the effect, that the disadvantage of being arranged across two walls diminished in Experimen3 in the corner-centered viewing condition. Future studies should try and disentangle this effect of an uneven distribution of pictures in a room on the viewing behavior and the memory of the presented stimuli.

Overall, the present results extend models on the influence of grouping principles on the processing of visual information^16^ to larger spatial environments. In addition, they fit well with the concept of partially hierarchical spatial mental models^4^. Applying McNamara's^4^ distinction between three kinds of spatial mental models to the present experiments would result in the following predictions (see also Table 2): Nonhierarchical models would predict an effect of distance between matching pairs but no effect of corner. Strongly hierarchical models predict the effect of corners and predict that distance would only have an effect if the matching pairs were on one wall. Partially hierarchical models predict both effects – namely, one of corner and one of distance.

**Table 2:** Model predictions and findings.

Predictions of models …	… According effect of subboundaries	… According effect of distance	Findings in three studies
Nonhierarchical models	−	+	Main effect of wall (experiment 1, 2, 3) contradicts this prediction
Partially hierarchical models	+	+	Main effects of wall and distance (experiment 1, 2, 3) support this prediction
Strongly hierarchical models	+	Within one subregion: + across subregions: −	Main effect of distance (Exp. 1, 2, 3) contradicts this prediction, as well as the significant effect of distance also for pairs across walls (single comparisons for interaction in Exp. 3)

The findings of these three studies were connected with predictions of mental models according to McNamara^4^.

First, the present findings add to McNamara's reaction time results^4^ findings on accuracy indicating that the spatial relationship between two cards was better remembered if they were shown on the same wall than if they were shown on two adjacent walls. This finding occurred even though the task required participants to explicitly ignore the boundaries. Their task was to remember matching pairs independently from their arrangement on one or two walls. However, region (i.e., wall) still played a crucial role, i.e. became part of the cognitive representation, as participants remembered matching pairs better if they were on the same wall. It seems that participants could not overcome the power of (visual) regions, although this was not conducive to the task at hand.

Second, the findings of the present study support partial hierarchical models because, in all three studies there were main effects for both wall and distance. Additionally, in Experiment 3 further findings also indicated partial hierarchical models. In single comparisons, the effect of distance became significant not only for matching pairs on one wall but also for matching pairs arranged across two walls, thereby contradicting predictions of strongly hierarchical models.

On the one hand, these observations are highly ecologically valid, because the experimental design simulated a typical everyday situation—namely, standing in a rectangular room and looking around on pictures on the walls. On the other hand, these conclusions must be taken with caution because the data were collected during the Covid-19 pandemic when participants took part online and used their own computer equipment and flat screens instead of experiencing an immersive Virtual Reality or a room in physical reality. However, the feeling of spatial presence can, but does not have to, depend on technical immersion^17^. For example, when memory performance in virtual and real environments was compared it was found that the effects were comparable^18^, however, the question of whether findings from virtual rooms can be transferred to real physical rooms still has to be examined further. Thus far, the findings of the reported experiments only concern information arrangements in virtual rooms perceived via two-dimensional displays. Nevertheless, the experimental situation used in the experiments came close to everyday perception: According to Gibson^19^ the basic visual properties depend on the viewpoint of an observer. For example, the same object seems to be smaller, if it is farther away or seems to be distorted if it is not plane in front of the viewer (according to the rules of a vanishing point). Gibson emphasizes, that these characteristics of everyday perception deliver important information to the viewer concerning the arrangement of objects in his or her surrounding environment. Of course, this ecological validity also means, that in the experiments 1–3, two pictures may show the identical picture of an everyday object, but the basic visual properties of the two cards depend on their arrangement within one wall or across two walls: If a viewer looked straight forward to the middle of a wall with two cards on this wall, these two cards were nearly identical in shape and only slightly distorted towards a vanishing point to the left and to the right. In contrast, if the viewer looked straight forward into a corner and the two cards were arranged one on the left and one on the right wall, the distortions toward a vanishing point were bigger in a graphical sense. So, one could argue, that the corner effect on memory is no effect of the existence of a corner between the two cards, but an effect of greater basic perceptual difference between the two cards in case of arrangement across two walls. But, this perceptual difference is based on the fact that the information is dispersed on different walls, and, introducing ecological settings like 3-dimensional rooms, this cannot be entangled, because perception in 3-dimensional rooms always underlies the laws of the vanishing point. If there would be no distortions of the form, the picture cards would simply not be perceived as hanging on the walls of a room.

Considering the influence of rooms on the viewing behavior of the participants, also visual attention should be taken into account: In literature^20,21^ the metaphor of a spotlight is used and it could be considered, if the walls and corners of a room not only influence the orientation of the visual field, but also the orientation of the attentional spotlight, i.e. the area in the visual environment where participants spent most of their attentional capacity. It can be assumed that two pictures forming a pair are more strongly connected during learning if they are in the attentional spotlight at the same time or if the attentional focus can switch faster or with less effort between the two if they are spatially near, and this could explain the distance effect: If there are three other pictures between the two pictures the probability, that these two pictures are in the spotlight at the same point of time or that the attentional focus can switch between the two easily, is smaller than if the two pictures are closer together, i.e. with only one more picture between them. But the size or shifting ease of the attentional spotlight cannot explain the corner effect found in Experiments 1–3, because the metrical distance between two pictures (e.g. with one other picture between them) was the same, independent if they were arranged within one wall or across two adjacent walls. The same was true for picture pairs with three pictures between them: The metrical distance was the same, independent of arrangement within one or across two walls. So, visual attention is a promising variable when examining the effect of arrangement on memory, but it cannot completely explain the findings in Experiments 1–3.

Additionally, to judge the influence of room corners on the cognitive processing of related visual information, further variables should be examined. The experiments in this paper focused on memory; however, for example, categorizing objects into sub- or super-categories could also be a process sensitive for the separating effects of corners^13^. Finally, further studies should consider individual differences such as spatial thinking or training in playing the classical children’s memory game.

The findings are of interest in all contexts in which observers are intended to mentally build connections between visual information units in physical or virtual rooms. Of course, in this respect, museums come into mind where curators invest much time in carefully arranging exhibits and putting them next to each other or on specific walls to support visitors in making connections between specific exhibits, thereby creating an intended scenography. The literature indicates that the educational message of an exhibition is clearly influenced by visitors’ movement through the museum space and that the kind of movement has an impact on the overall impression of the exhibition^22^. The findings reported here suggest that also the way in which exhibits are arranged on museum walls and the way in which visitors explore those rooms might influence not only an overall impression but also the concrete connections between single specific exhibits that viewers perceive and construct. This might also hold for other spaces where information is presented and informal learning may occur as, for example, in zoos or historical sites. In light of these findings, retail shops should also consider which articles to present next to each other on one wall and which articles to separate by a corner, even if the metric distance between the articles remains the same. Furthermore, the increased usage of virtual three-dimensional environments on the internet when presenting information to users, with their freedom to create rooms and arrange objects within, makes research on the corner effect found in this paper interesting: Arranging visual information in a virtual three-dimensional room not only “brings information to life” and shapes the overall impression but also influences the organization of memory. Finally, it must be considered that not only does the environment at hand have an effect on users and their cognitive processes, but also that this effect is driven by the everyday viewing behavior of the users, namely, perceiving rooms in a wallwise manner. That may open the field for designers not only to consider how to shape rooms and how to arrange information within them, but maybe also to take into account users everyday viewing behavior.

## Method

This project was supported by the institute’s local ethics committee, #LEK 2020/012, which commits itself to adhering to the rules on safeguarding good scientific practice laid down in the “Guidelines for Good Scientific Practice in the Leibniz Association” and the “Guidelines for Safeguarding Good Research Practice. Code of Conduct” of the Deutsche Forschungsgemeinschaft (DFG). The procedures and templates of the committee are based on the recommendations of the Deutsche Gesellschaft für Psychologie (DGPs)^23^. It is confirmed that all methods were carried out in accordance with relevant guidelines and regulations. It is also confirmed that all experimental protocols were approved by the institute’s local ethics committee.

Each experiment started with information on the procedure, and the participants provided informed consent. The studies and their measurements and analyses were outlined before the data were collected on the AsPredicted platform (Exp. 1: #53,821, Exp. 2: #58,138, Exp. 3: #90,942).

### Experiment 1: the corner effect on memory

#### Participants

Participants were recruited using the online participant recruitment system Prolific (https://www.prolific.co). Because the interesting hypothesis of all three experiments was, how the arrangement of picture pairs within one wall versus across two adjacent walls influences memory, the number of participants was calculated based on a main effect and not an interaction. Therefore, it is possible, that interactions were underpowered. A prior study (preregistered at AsPredicted #45,012) with 22 participants revealed a significant effect of "wall" on sensitivity (η_p_^2^ = 0.38). Based on this value, G-Power^24^ suggested a sample size of n = 25 to detect a significant within-participant effect with a power of 0.95 at the alpha level of 0.05. Because online studies are prone to disturbances, data from n = 30 participants were collected. Thirty participants started the study on Prolific and completed the whole experimental procedure. Before any data analysis, the data of one participant were excluded because the log file showed that the experimental session of this participant took extremely long with pauses not only between but also within blocks. Another dataset from one person was deleted because at the end of the experimental session the participant did not mark the box next to the statement that would allow the data to be used for research. The data of the resulting 28 participants were then analyzed. These 28 participants were aged 18–58 years (M = 32,64), 8 were female and 20 were male.

#### Stimulus material

The stimulus material was programmed using the game engine Unity, and the picture cards were taken from THINGS, a database with more than 26,000 naturalistic object images^25^. Each participant was presented nine different virtual rooms in randomized order. Each room was four sided and displayed pictures on three walls and had a virtual door on the fourth wall (see Fig. 1). On every wall with pictures, 12 pictures were arranged in two rows of six pictures each. The first room served as the practice room,the following eight rooms were used for experimental data acquisition. Each room presented pictures of everyday objects from one single category: The practice room always presented sports objects; the other eight rooms presented pictures of objects from the categories sweets, vegetables, fruits, clothing, home keeping, toys, vehicles, or animals. The order of the room categories was randomized across participants. Each room had 18 picture pairs. Thus, every picture occurred twice, and two cards showing the same picture constituted a matching pair with the two cards showing the identical photo of an object. The two pictures forming a pair were arranged either on the same wall or on two adjacent walls. Between the two pictures, there was either one or three other pictures. The pictures constituting an experimental pair were randomized, i.e., across participants, for example, the pair of apples was in every condition. Pictures of pairs in the test trials, i.e. trials that later were used for data analyses, were always arranged in the same row. To prevent participants from memorizing the pictures only in a “rowwise manner”, in every room, four pairs were also displayed with the two pictures arranged in different rows (namely, one picture in the upper row and one picture in the lower row); these pairs (filler trials) were not analyzed. All other pictures were arranged randomly.

Overall, this resulted in a 2 (distance: 1 vs. 3 pictures between pictures of a pair) × 2 (wall: pictures of a pair arranged on one vs. across two walls) factorial within-subjects design.

#### Procedure

The experiment was conducted online; that is, the participants used their own laptops or computers, but no handhelds, such as cell phones or tablets. The virtual rooms were programmed as virtual three-dimensional rooms and were displayed on the participants’ laptop or computer screens.

After providing informed consent, the participants read an introduction describing the steps of the experiment, the virtual rooms, and their main task, namely, remembering pairs of cards with the same picture.

In each room, there was a learning and test phase (see Fig. 1). When the participants started a new room, they were placed virtually on the floor in the middle of the room with the virtual door at their back, that is, facing the front picture wall with one picture wall to the left and one to the right. During the experiment, they could not move around in the room. The participants could only turn their view around 360 degrees by using the left and right arrow keys. In each room (also in the practice room at the beginning), participants followed the same procedure. First, there was a learning phase: by pressing the left and right arrow keys, participants could freely look around in the room and were asked to memorize the arrangement of the picture pairs for two minutes.

Then, in the same virtual room, a test phase followed: All cards were turned around so that only their blank backs were visible. There were twelve trials in each room. Every trial started with an orange line on the floor. Participants were asked to orient their field of view in the direction of this line by using the arrow keys. When their field of view was oriented in this direction, two cards in their field of view became important: one card was turned so that the picture of the card became visible, and the other card (still with only the blank back visible) was marked by an orange frame. Now the participant had to decide whether the card with the orange frame would show the same picture when turned around (i.e., if the two cards were forming a pair) or whether the two cards would show different pictures by pressing one of two keys (“A” or “S”; see Fig. 1). In every room, a participant worked through twelve trials (eight test trials and four filler trials) in randomized order. Of the test trials, four trials showed cards arranged on one wall, and four trials showed cards arranged across two walls. One half of them had one other card between them, and the other half three cards. Furthermore, for each wall and distance combination, one trial was considered “true” (same picture) and one trial was considered “false” (different pictures). Additionally, in each room, four filler trials were tested but not analyzed. These filler trials involved asking for two cards arranged in different rows.

After working through all twelve trials in a room, the participants saw a text on a gray background asking them whether they wanted to proceed to the next room or to have a little break. If they wanted to go on, they started the next room by pressing the space bar. Then the next room was visible, presenting pictures with everyday objects belonging to another category.

Two kinds of data were collected: (1) After each trial, it was recorded whether a participant hit the “pair” or “no pair” button. (2) In each room during the learning phase, every 0.5 s, the direction in which the participant oriented his or her central field of view was recorded. This was measured at 360 degrees, with 0 degrees being in the middle of the front wall (the wall across from the door) and then in the clockwise direction; the corner, for example, the right corner near the front wall was 45 degrees and the middle of the right wall was 90 degrees (see also Fig. 3).

#### Results

##### Sensitivity

The data of 28 participants were analyzed according to the signal detection theory^14^. First, for every participant, his or her nonparametrical overall sensitivity A’^15^ was calculated. A’ measures sensitivity like the more common measure of d’, but without making the assumptions that (1) the signal and noise distributions are both normal and (2) the two distributions have the same standard deviation. An A’-value of 0.5 indicates that signals cannot be distinguished from noise, and an A’-value of 1 indicates perfect performance. According to Stanislaw and Todorov^15^, values of A’ < 0.5 may arise from sampling errors or response confusion. One participant had an overall sensitivity of A’ < 0.46. The memory data of this participant were excluded. Analyses of sensitivity were therefore based on the data of 27 participants and descriptive analyses of viewpoints on the data of all 28 participants.

##### Field of view orientation

During the learning phase, every 0.5 s, the participants’ central viewing direction was measured. The viewing direction straight ahead to the middle of the wall across the door was defined as 0 degrees, and 360 degrees were defined in horizontal clockwise direction. For example, a viewing direction straight to the corner to the right was 45 degrees, and a viewing direction to the middle of the wall to the right was 90 degrees. The 28 participants provided 53,760 central viewing directions (28 participants × 8 rooms × 2 min). All viewing directions during the first 5 s in a new room were excluded because all participants started in each room with a viewing direction of 0 degrees because this direction is the ecologically valid viewing direction when stepping into a new room through a door. Additionally, all viewing directions during which a participant pressed an arrow key were excluded since the viewpoint was moving during these time intervals. Therefore, a total of 9726 views were excluded (18.1%), and 44,034 views were analyzed. Figure 3 displays the number of central viewing directions at intervals of 10 degrees. As can be easily observed, central viewing directions were not evenly distributed across a room.

### Experiment 2: replicating the corner effect and examining lines

#### Participants

Participants were recruited using the online participant recruitment system Prolific (https://www.prolific.co). Based on the partial eta square = 0.21, G-Power^24^ suggested a sample size of 52 participants. Because online experiments are especially susceptible to disturbances, we planned to collect data from 55 participants. The data of the participants who did not complete the whole experiment or who reloaded the experiment in between (thereby making several trials double) were not analyzed. Therefore, only the data of the resulting 51 participants were then analyzed. Sixteen of these participants were female and 35 were male,two of them did not reveal their age. The age of the remaining 49 participants ranged from 18 to 58 years (M = 29.16).

#### Stimulus material

Experiment 2 was similar to Experiment 1 with one exception: one more kind of room was introduced, namely, a room with a round wall, i.e., a rotunda, without any corners but instead with vertical lines separating areas of the (one) round wall (see Fig. 1). The room was divided into four areas by displaying four perpendicular lines with equal distances from each other. On one area, there was a door, and on the other three areas, visual information was again presented. Therefore, the special characteristic of the rotunda room is that it is three-dimensional but has only one wall, which is curved and surrounds the whole room. Because of the manipulation with the four perpendicular lines, this curved wall is divided into four areas. For simplicity, these four areas of the rotunda room are also referred to as “four walls”. Additionally, to replicate the findings of Experiment 1, the box-like three-dimensional room used in Experiment 1 with the four sided floor was used, again with a door on one wall and visual information on the other three walls. In addition to the round versus q format of the floor, the only difference between the two rooms were the perpendicular lines: Whereas in the rotunda, there were explicit perpendicular gray lines dividing the room into four areas, in the box room there were no lines but only the corners that divided the four walls from each other. The circumference and height were the same in the rotunda room and the box room. Again, as in Experiment 1, in each room, 18 picture pairs were arranged. Overall, this procedure resulted in a 2 (distance: 1 vs. 3 pictures between pictures forming a pair) × 2 (wall: pairs arranged on one vs. across two walls) × 2 (architecture: box-like room vs. rotunda) factorial within-subjects design.

#### Procedure

Again, Experiment 2 was conducted as an online study via Prolific. The participants used their own desktop PC or laptop. The procedure was similar to that of Experiment 1, with one exception: The participants had to work through ten instead of nine different rooms. The first two rooms were used for training, and all the images on the walls showed objects from the sports category. One of these two training rooms was a box room and the other was a rotunda room. The next eight rooms were experimental rooms and were used for data gathering. Four of them were box rooms and the other four were rotunda rooms, in randomized order. The content of each room, the set of picture pairs, and their arrangement on the walls were similar to those in Experiment 1.

#### Results

##### Sensitivity

The data of 51 participants were analyzed according to the signal detection theory^14^. First, for every participant, his or her nonparametrical overall sensitivity A’^15^ was calculated. None of the participants had an overall A’ < 0.5,therefore, the data of all 51 participants could be analyzed.

##### Field of view orientation

As in Experiment 1, during the learning phase, every 0.5 s the participants’ central viewing direction was measured. The 51 participants provided 97,920 central viewing directions (51 participants × 8 rooms × 2 min). Again, all viewing directions during the first 5 s in a new room were excluded, as was all viewing directions during which a participant pressed an arrow key. Therefore, a total of 20,256 views were excluded (20.69%), and 77,664 views were analyzed. Figure 3 displays the number of central viewing directions in steps of 10 degrees. As can easily be seen, again, central viewing directions were not evenly distributed across a room.

### Experiment 3: controlling the orientation of the central field of view during learning

#### Participants

Participants were recruited using the online participant recruitment system Prolific (https://www.prolific.co). An analysis with G-Power^24^ based on the average effect size found in the former experiments suggested a sample size of 42 participants. Fifty-four participants started the study on Prolific, but eight of them did not provide a complete dataset and were excluded from any analyses. The data of the resulting 46 participants were then analyzed. These 46 participants were aged 18–49 years (M = 28.65), 23 were female and 23 were male.

#### Stimulus material, procedure and design

The stimulus material, procedure, and design were the same as those in Experiment 2 with only two exceptions: (1) Participants only entered box like rooms, no rotunda rooms. (2) During the learning phase, participants could not freely turn around but had fixed viewing directions. In the learning phase, they could only shift their central field of view in steps of 90 degrees by using the left and right arrow keys. In one half of the rooms, the central viewing direction was oriented toward the middle of the walls, and in the other half of the rooms, the central viewing direction was oriented toward the corners (see Table 1). At the beginning of each session, there were two training blocks: one with the central field of view toward walls and one toward corners. In the learning phase, the order of the two viewing directions in the experimental rooms was randomized for each participant. This resulted in a 2 fixed viewing direction (learning perspective centered toward walls/corners) × 2 distance (1/3 pictures between the pictures forming a pair) × 2 wall (pairs arranged on one wall/across two walls) within-subjects design.

#### Results

##### Sensitivity

The data of 46 participants were analyzed according to the signal detection theory^14^. First, for every participant his or her nonparametrical overall sensitivity A’^15^ was calculated. None of the participants had an overall sensitivity of A’ < 0.5 and therefore the data of all 46 participants could be analyzed.

## Data Availability

The datasets generated and analyzed during the current studies are available in the OSF repository, 10.17605/OSF.IO/PKHV3.

## References

[1] Annaud, J.-J. *The Name of the Rose*. Produced by Bernd Eichinger (1986).

[2] Palmer SE (1992). Common region: A new principle of perceptual grouping. Cognit. Psychgol..

[3] Montoro PR, Villalba-Garcia C, Luna D, Hinojosa JA (2017). Common region wins the competition between extrinsic grouping cues: Evidence from a task without explicit attention to grouping. Psychon. Bull. Rev..

[4] McNamara TP (1986). Mental representations of spatial relations. Cogn. Psychol..

[5] Choi H-H, van Merrienboer JJG, Paas F (2014). Effects of the physical environment on cognitive load and learning: Towards a new model of cognitive load. Edu. Psychol. Rev..

[6] Meyers-Levy J, Zhu RJ (2007). The influence of ceiling height: The effect of priming on the type of processing that people use. J. Consum. Res..

[7] Roberts AC (2019). The cubicle deconstructed: Simple visual enclosure improves perseverance. J. Environ. Psychol..

[8] Wu Y (2021). Rounded or angular? How the physical work environment in makerspaces influences makers’creativity. J. Environ. Psychol..

[9] Radvansky GA, Copeland D (2006). Walking through doorways causes forgetting: Situation models and experienced space. Mem. Cognit..

[10] Chan J, Nokes-Malach TJ (2016). Situative creativity: Larger physical spaces facilitate thinking of novel uses for everyday objects. J. Probl. Solving.

[11] Hund AM, Plumert JM (2003). Does information about what things are influence children’s memory for where things are?. Dev. Psychol..

[12] Plumert JM, Franzen LJ, Mathews MM, Violante C (2017). Linking, “what” and “where” information: How the strength of object categories influences children’s memory for object locations. J. Exp. Child Psychol..

[13] Schneider IK, Mattes A (2022). The effect of spatial distance between objects on categorization level. Psychon. Bull. Rev..

[14] Macmillan NA, Creelman CD (2005). Detection Theory: A User’s Guide.

[15] Stanislaw H, Todorov N (1999). Calculation of signal detection theory measures. Behav. Res. Methods Instrum. Comput..

[16] Beck DM, Palmer SE (2002). Top-down influences on perceptual grouping. J. Exp. Psychol. Hum. Percept. Perform..

[17] Wirth W (2007). A process model of the formation of spatial presence experiences. Med. Psychol..

[18] Arthur EJ, Hancock PA, Chrysler ST (1997). The perception of spatial layout in real and virtual worlds. Ergonomics.

[19] Gibson, J. J. *Wahrnehmung und Umwelt*. München, Wien, Baltimore: Urban & Schwarzenberg. ISBN 3541099313 (1982).

[20] Posner MI (1980). Orienting of attention. Q. J. Exp. Psychol..

[21] Eriksen CW, Yeh YY (1985). Allocation of attention in the visual field. J. Exp. Psychol. Hum. Percept. Perform..

[22] Wineman JD, Peponis J (2010). Constructing spatial meaning: Spatial affordances in museum design. Environ. Behav..

[23] Deutsche Gesellschaft für Psychologie, DGPs (Ed.) *Ethisches Handeln in der psychologischen Forschung: Empfehlungen der Deutschen Gesellschaft für Psychologie für Forschende und Ethikkommissionen* (Göttingen, Hogrefe, 2018). ISBN 9783801728021.

[24] Faul F, Erdfelder E, Lang AG, Buchner A (2007). G*power 3: A flexible statistical power analysis program for the social, behavioral, and biomedical sciences. Behav. Res. Methods.

[25] Hebart MN (2019). THINGS: A database of 1,854 object concepts and more than 26,000 naturalistic object images. Plos One.

